# Snap4City Platform to Speed Up Policies

**DOI:** 10.1007/978-3-030-57764-3_7

**Published:** 2020-09-11

**Authors:** Nicola Mitolo, Paolo Nesi, Gianni Pantaleo, Michela Paolucci

**Affiliations:** 1 EURAC Research, Bolzano/Bozen, Italy; 2 EURAC Research, Bolzano/Bozen, Italy; 3 Faculty of Environment and Technology, UWE, Bristol, UK; 4 European Commission, Joint Research Centre (JRC), Ispra, Italy; DISIT Lab, DINFO, University of Florence, Florence (Firenze), Italy

**Keywords:** Big data architecture, Smart City Control Room, Decision-support system, Advanced APIs, IoT

## Abstract

In the development of smart cities, there is a great emphasis on setting up so-called Smart City Control Rooms, SCCR. This paper presents Snap4City as a big data smart city platform to support the city decision makers by means of SCCR dashboards and tools reporting in real time the status of several of a city’s aspects. The solution has been adopted in European cities such as Antwerp, Florence, Lonato del Garda, Pisa, Santiago, etc., and it is capable of covering extended geographical areas around the cities themselves: Belgium, Finland, Tuscany, Sardinia, etc. In this paper, a major use case is analyzed describing the workflow followed, the methodologies adopted and the SCCR as the starting point to reproduce the same results in other smart cities, industries, research centers, etc. A Living Lab working modality is promoted and organized to enhance the collaboration among municipalities and public administration, stakeholders, research centers and the citizens themselves. The Snap4City platform has been realized respecting the European Data Protection Regulation (GDPR), and it is capable of processing every day a multitude of periodic and real-time data coming from different providers and data sources. It is therefore able to semantically aggregate the data, in compliance with the Km4City multi-ontology and manage data: (i) having different access policies; and (ii) coming from traditional sources such as Open Data Portals, Web services, APIs and IoT/IoE networks. The aggregated data are the starting point for the services offered not only to the citizens but also to the public administrations and public-security service managers, enabling them to view a set of city dashboards ad hoc composed on their needs, for example, enabling them to modify and monitor public transportation strategies, offering the public services actually needed by citizens and tourists, monitor the air quality and traffic status to establish, if impose or not, traffic restrictions, etc. All the data and the new knowledge produced by the data analytics of the Snap4City platform can also be accessed, observing the permissions on each kind of data, thanks to the presence of an APIs complex system.

## Introduction

In most of the modern smart cities, there is a great emphasis on setting up the so-called Smart City Control Room (SCCR) that is an area in which all the data are collected and aggregated and where high-level data/results are summarized and made accessible for decision makers and city operators. In large metropolitan cities, the SCCR includes large panels/monitors on which the status of the city is displayed in real-time presenting predictions and alerts regarding: mobility, energy, social activities, the environment, weather, public transportation, people flow, health, water, security, ICT, governmental, first aid, civil protection, police, fire brigade, hospital triage, and thus almost all the city resources expressed via key performance indicators (KPI). Some of the city’s monitored resources are representative of critical infrastructures such as: mobility and transport, energy, security, health, water, civil protection, governance, ICT, etc. In medium-sized cities, the daily management of city resources is performed by a set of city operators, which could be legally independent with respect to the central municipality and should autonomously manage their SSCR (Azzari et al. 2018). To realize a SCCR, it is necessary to understand: (i) the SCCR requirements; (ii) data sources needed to monitor the scenarios; and (iii) services the decision makers want to provide to various categories of users. One of the key aspects consists of establishing relationships that define guidelines and common operating modes with all the stakeholders and operators who have to work together to create/manage the dashboard (DeMarco et al. 2015; McArdle et al. (2016); Suakanto et al. 2013). In this regard, it is of fundamental help to use collaborative tools such as the Living Lab support on Web portals. In the context of the EC REPLICATE project, the City of Florence has set up a SCCR solution based on Snap4City technology (https://www.snap4city.org) by collecting and aggregating a relevant amount of data, performing data analytics to provide predictions, early warning, routing, heat maps, and a large set of dashboards for the SCCR. This paper is organized as follows: Sect. 2 reports the process of the adoption of the SCCR, including Living Lab support; in Sect. 3, the Snap4City architecture is described; Sect. 4 provides a scenario of data collected and aggregated for the SCCR and related services; Sect. 5 reports some statistics regarding the usage of SCCR in the case of Florentine area. Conclusions are drawn in Sect. 6.

## Adoption Process

**Fig. 1:**
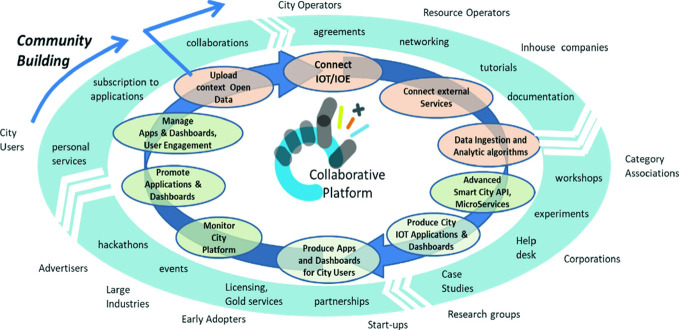
Snap4City collaborative workflow process

Smart cities are complex ecosystems in which many distinct aspects coexist, and many kinds of actors interact. Living Labs (LL) are instruments for the development and implementation of technology to accelerate innovation in cities (Villanueva-Rosales et al. 2016; Coenen et al. 2014). A LL is a starting point to collaborate on and generate models to create smart cities (Cosgrave et al. 2013; Majeed et al. 2017; Concilio 2016), i.e., it is a way to develop collaborative systems to engage the community/stakeholders (e.g., students, lecturers, computer scientists, electronics engineers, politician and tourists). Snap4City provides a set of tools and solutions to quickly setup and then maintain a LL. The approach has been realized involving different kinds of organizations (universities, SME and large industries and public administrations) and users (city and resource operators), in-house companies (company participated by the city body), tech providers, associations, corporations, research groups, start-ups, early adopters, large industries, advertisers, city users, community builders, etc.), thus reflecting the features described in quadruple helix (QH) (Arnkil et al. 2010; Alba M et al. (2016); Karvonen et al. 2018), to support the LL concept in smart cities (see Fig. 1), also considering a multi-organizational/multicity approach that enables the sharing of experiences among cities.

At the same time as the setup is done, the collaboration among stakeholders can start by creating: agreements, collaborations, networking, tutorials, workshops, hackathons, etc. (see Fig. 1), so as to arrive at involving all the stakeholders in concrete smart cases, signing agreements, partnerships, licensing, etc. This process should be driven by the municipality, which may need support for technical aspects that are provided by Snap4City at different levels. Typically, the single companies, even if supported by the city/operators, do not have the perspective and the mission to put in common so large a multi-domain multiservice framework and environment.

## Snap4City Architecture

**Fig. 2:**
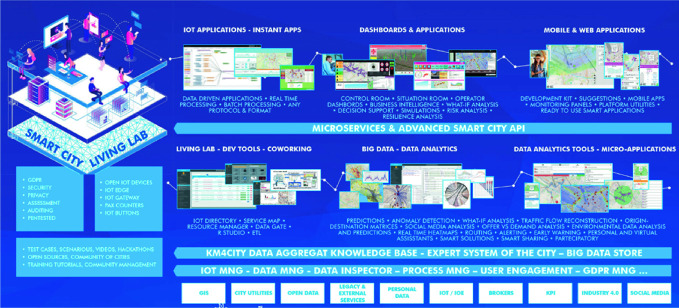
Snap4City architecture and ecosystem

Most of the smart city solutions must cope with big data volumes, variety and veracity (Bellini et al. 2014). Open data as static data (street graph, point of interest, etc.) are not the only data sources needed for the city SCCR: most of the big data ingested and managed periodically or in real time comes from public transport/vehicle and human mobility in city, events, energy, governance, IoT, etc. A smart city architecture should be capable of taking advantage of a huge amount of data coming from several domains, at different speed/rates for exploiting and analyzing them, for computing integrated and multi-domain information, making predictions, detecting anomalies and early warning, and for producing suggestions and recommendations for city users and operators. In this sense, the Snap4City architecture and the integrated semantic model, connected to the Km4City multi-ontology (Bellini et al. 2014), have been developed to collect, aggregate and manage these different kinds of data (see Fig. 2) to provide a set of services: dashboards, smart decision-support systems; social media-monitoring Twitter vigilance; statistics and prevision analysis, suggestions and recommendations for citizens, etc.

Snap4City consists of a set of modular tools accessible according to the user profile:

- **data registration, ingestion and publication**. Data connections are typically bidirectional. They can come from/to: (i) any kind of data source/collector, protocol and format from/to: open data, real-time data, GIS, IoT data, data stream, data driven, social media, Industry 4.0, etc.; (ii) IoT devices and networks, IoT edges, gateways, IoT brokers, via various protocols and formats; (iii) Web-scraping processes; (iv) dashboards collecting actions from the users via: buttons, dimers, time selectors, and showing them; (v) mobile applications collecting and presenting; (vi) results of data analytics, including personal data produced by users (Nesi et al. 2018).
- **Storing and indexing of data** are performed via the Km4City smart city ontology and knowledge base on graphDB (Bellini et al. 2014); and big data store for data shadow, based on Elastic Search and/or Hbase noSQL database. The spatial, temporal and semantic indexes are created for fast and smart data retrieval with capabilities of inference, reasoning, faceted search, drill-down and extracting insights from the data and contexts.
- **Advanced Smart City API and Microservices** for accessing services and data. All the data collected and indexed can be accessed via APIs and/or Microservices to enable developers and operators to create in a simple manner: IoT applications, mobile and Web app, and dashboards, according to the GDPR (Badii et al. 2019).
- **Data analytics and data transformations processes and tools** can be created and shared as the smart core of the smart services, such as: making predictions, signaling early warnings, detecting anomalies, creating analysis, producing interactive heat maps, suggesting decision makers, supporting simulations, etc. Analytics and data processing exploit Smart City API, can be developed in R, Python, Java, IoT App Node-RED, ETL, etc., and can be executed on demand/on events, periodically and in real time. Resulting data are also saved and indexed into the platform.
- **Dashboards** can be created for various kinds of users, such as: the major decision makers, city operators, ICT operators and private users. They can be suitable for SCCRs with large video walls, large control rooms with tens of operators on desktops of multiple monitors, mobile operators and situation rooms with touch panels, as well as for virtual control room in which the controls are distributed and views are distributed among various locations.
- **IoT applications** are data-driven and/or periodic visually defined data-flow processes exploiting the suite of Snap4City Microservices (more than 150 nodes). They can be put in execution IoT edge, as well as on Cloud, and are based on Node-RED. IoT app also enables to perform business logic **(**i.e., the logic behind the applications which can be used to enforce the business rules and the application “intelligence”), smart interaction and data transformation behind the Snap4City dashboards, integrating ticketing, video wall dynamic re-configuration, etc. Microservices may also manage processes for data analytics, Web scraping, external services, data gathering and publication, etc. (Badii et al. 2018).
- **Web and mobile applications** can be created by developers exploiting Advanced Smart City APIs. The app may be managed by specific Snap4City tools to send engagements, surveys, stimulus and for understanding user behavior, creating origin-destination matrices, getting reactions from the users, informing of critical conditions, etc. (Badii et al. 2017a).
- **Living Lab support** makes possible creating a collaborative sustainable environment for smart city growth in which the city stakeholders can contribute, according to their skill and commitment. Snap4City provides an environment in which Living Lab users can exchange information, work on collaborative tools for data transformation and valorization, exploiting all the other tools.

The whole platform is GDPR-compliant and enforces privacy and security for data, dashboards, IoT devices, IoT applications, personal data, data analytics and processes, etc., which can be private or can be delegated to pass the ownership to other users.

## Data Versus Services

**Fig. 3:**
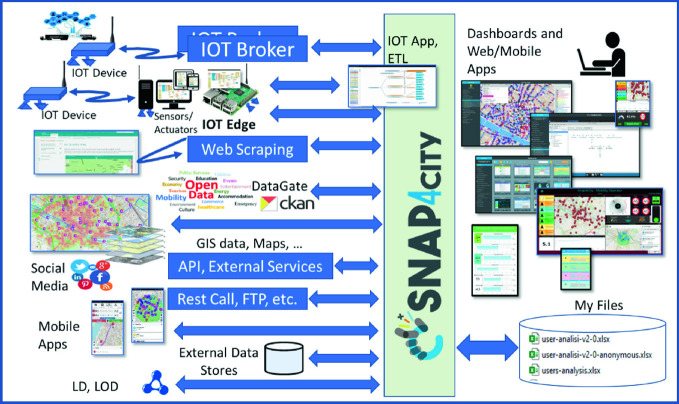
Snap4City: data ingestion and final services

The kind of services provided to the final users are strictly connected to the data in/out activities and the interaction among the data providers. Each data provider can have a different modality to expose, transmit and receive data, moreover the data can be formalized in various modalities (structured or non-structured information), formats (csv, Excel, XML, json, etc.), and protocols in push and/or pull, etc. This is the reason why a smart city big data platform, such as Snap4City, must be flexible and capable to ingest every various different kinds of data (see Fig. 3).

Once the data have been collected and integrated, the historical data and real-time data can be exploited by data analytics processes, IoT apps, dashboards and apps. The original data and results of the data analysis can be accessed for business intelligence tools and SCCR. The most relevant data available in the Florence SCCR, are described next and classified based on their context:

- **Mobility**: public transport operators schedule and paths, traffic-flow sensors, parking status, cycling paths, road graph, accidents and traffic events, ordinances, car-park occupancy prediction, what-if routing, quality of public transportation services, etc. The services produced, starting from those data are: KPI assessing quality of public transportation; routing and multimodal routing; real-time traffic-flow reconstruction (Bellini et al. 2018, results of the Sii-Mobility project https://www.sii-mobility.org); real-time status and predictions for parking, smart parking;
- **Environment**: (i) irrigators; (ii) smart waste; (iii) air quality sensors: PM10, PM2.5, CO, benzene, NO, NO_2_, O_3_, temperature, humidity, etc.; (iv) air quality heat maps for pollutants at point (iii); and (iv) pollination. As derived data, we can have NO_X_ predictions (as a result of the TRAFAIR CEF project, https://trafair.eu); air quality heat maps updated every hour, real-time status and trends related to air quality sensors and pollination, NO_*X*_ predictions for the next 48 h, with a resolution of 4 × 4 m and at two levels of height (at 3 and 6 m); and long-term predictions for pollutants’ critical days;
- **Energy**: (i) recharging stations (fast and regular); (ii) consumption meters (smart info); (iii) smart light; monitoring consumption via smart meters, smart lights status and trends;
- **Weather**: forecast and actual (related to temperature, wind, clouds, sun, rain, etc.);
- **Social**: (i) smart benches; (ii) entertainment events; (iii) Twitter monitoring via Twitter vigilance (https://www.disit.org/tv), sentiment analysis, NLP text; (iv) TV-camera streams; and (v) triage status of major hospitals; also for early warning.
- **People Flows**: (i) Wi-Fi status; (ii) origin-destination matrices, people-flow analysis (as result of the EU RESOLUTE project, https://www.resolute-eu.org) from the Wi-Fi network of access points;
- **Governmental and Communications**: (i) KPI of the city, including COVID-19 data; (ii) digital signage (not directly included into the solution for Florence); (iii) civil protection, resilience guidelines and suggestions, KPIs (RESOLUTE project);
- **Tourism and Culture**: Points of interests (POI), cultural events, etc.;
- **Analysis**: (i) what-if routing; (ii) scenarios; (iii) traffic flow; and (iv) environmental predictions.
- **Video streams**: (not directly included into the Snap4City solution for Florence).

Starting from these kinds of data, reworking them (semantic aggregation in compliance with Km4City multi-domain ontology and data analytics) and thanks to the APIs presence, a set of services is available in the Florence SCCR. The series of dashboards designed to provide the services just described, a mobile app was created and called ‘Florence where what…’ (see next section) and are capable to send suggestions and recommendations based on location and the preferences expressed by citizens and tourists.

## Smart City Control Room

A SSCR is a solution and structure in which all data and indicators of the city are collected and aggregated to produce summary visions, indicators, forecasts, precursors, anomaly indicators, etc. The results are produced through big data analytics to support decision makers and operators, so that they can quickly understand the situation in full and act, also through simulations for scenarios with what-if conditions. When the city grows, its systems become more complex. Moreover, some cities are morphologically complex during their histories, morphogenesis and structures, and it becomes important to:

- **manage the data** collection and integration, data analytics, prediction indicators (predictions, early warnings, anomalies), but also to carry out simulations and comparing them with the hypotheses on events, realized before the simulation and without the Snap4city tools.
- **Activate** and run data-analytic algorithms that can produce systematic or, if necessary, real-time forecasts, identifications of anomalies, and the ability to communicate them to operators and from an early warning and study perspective. Therefore, they are able to generate reports even in advance.
- **Visualize** the state of the city and its evolution and critical aspects for the different operators (in a common operating room, as in the situation rooms), allowing also some remote operators in their offices to access some summary information, prediction, service status, etc.
- **Enable the carrying out**, directly on the dashboards, the necessary **in-depth analysis** with drill-down techniques (time space and for reports), with: What-if simulations on problems and solutions, routing algorithms, predictive models and in-depth analysis tools. On these, it must be possible to **open discussions/chats** with other operators, even remote ones (via radio, voice and chat), and to bring the attention of all operators to support decisions.
- **Manage events and reports** that may arrive from various operators: mobility, transport, waste, energy, social media, highways, public transport, etc., in various standards and through various communication channels. Managing in these cases means: coordinating possible joint actions between several operators, acting, following their evolution, and keeping track of events, until their conclusion/resolution, to take them into account for the next actions.

### A Set of Connected Views and Tools

The SCCR is a decision-support tool that is able to provide evidence of the arrival/occurrence of critical conditions in real time and/or precursors of such conditions and offer solutions (decision support, e.g., by proposing multiple choices on evacuation plans, routing and activation of implementation plans). Following these guidelines, the Florence SCCR has been realized with a series of ad hoc dashboards connected each other:

- **Main dashboard**, ‘Firenze Oggi’ (Florence Today), contains a set of KPIs monitored in real time: a number of users connected to the public Wi-Fi, civil-protection messages, weather forecasting, recharging stations statuses, state of road maintenance and accidents, public transport lines; but also statistical data such as: census numbers (births, deaths, marriages and civil unions) and analyses of: traffic, pollution and what-if analysis. It has links to the following dashboards.
- **Energy**: position on the city map, real-time status and historical trends of: fast and normal recharging stations, ZTL gates, Wi-Fi, smart irrigators, smart lights, statistics on residential smart energy meters. KPIs: monthly cumulative energy consumption, average weekly consumption of each fast recharging station, number of users connected to the mobile app, eEnergy consumption via mobile app, accumulator status (used), etc.
- **Environment**: position on the city map, real-time status and historical trends of: air quality stations and low-cost sensors (PM10), weather sensors, pollen monitoring, Florence weather for today and for the next two days (clouds, rain, sun, wind), temperature, waste sensors, etc.
- **Mobility**: traffic events in Florence, traffic daily inflow/outflow trend, total number of inflow/outflow vehicles, car-park statuses and trends, etc.
- **Social**: Twitter sentiment trends in the city of Florence, natural-language processing and sentiment analysis on tweets (most used hashtags, statistics on Twitter citations), people flow.
- **Resilience**: civil-protection messages in real time, hospital first aid, evacuation paths, etc.

**Fig. 4:**
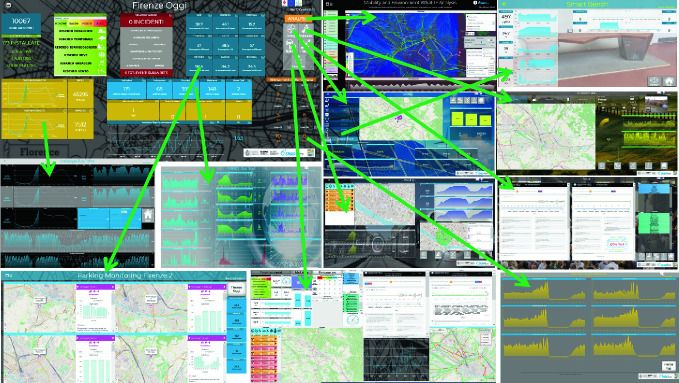
Florence Smart City Control Room: connected views and tools

Moreover, other additional views can be opened. In Fig. 4, some of the connections are shown. Starting from the mobility dashboard and clicking on ‘traffic daily inflow/outflow trend,’ it is possible to see details regarding the traffic flow: statistic and trends, ZTL, comparison among actual and past values (Badii et al. 2017b).

## Usage and Validation

**Fig. 5:**
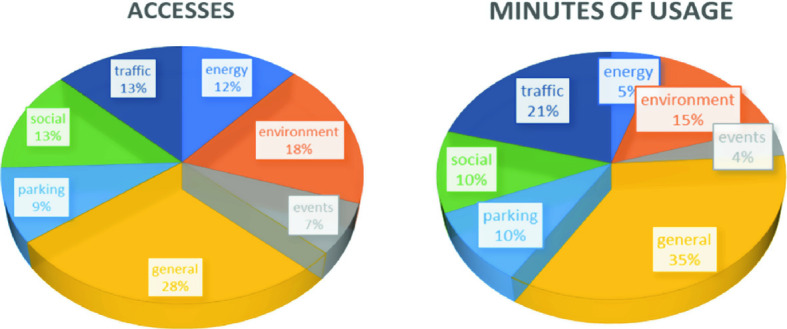
View on the usage of the dashboards related to the major topics for the area of Florence

The Florence SCCR development started on 2017 with the first dashboard, and since that time, the number of data has been increased with corresponding views and dashboards of various kinds. Most of the data are public and accessible on dashboards from the Web site of Snap4City.org, while a number of dashboards are private to the municipalities. In Fig. 5, we have reported an aggregated view of the usage and attention that dashboards have received from the users. The views are classified basing on number of accesses and minutes of usage for the 2019. The dashboards describing the general view and the traffic aspects were the ones on which users spent the most time on viewing. While the dashboards that have received the most access are those related to general and the environment aspects. To measure how those data have influenced the policies of the municipality is not addressed, while it is evident which are the major topics of interest in the area.

The attention on general aspects, mobility and environment are a confirmation of the effective usage and need of an instrument such as an SCCR for monitoring the city. In summary, a total of more than 6000 accesses have been made and 340,000 min have been spent on the major dashboards for the major areas in the year 2019, which is an average of 57 min per access. On the other hand, there are dashboards that are typically accessed 24H/7D, and those that are only sporadically used.

## Conclusions

Smart City Control Rooms, SCCRs, are getting strong attention at this time for their capability of reporting in real time the status of several city aspects, thus creating a strong and effective tool for decision makers. This paper presented Snap4city as a Big Data Smart City Platform to support the city decision makers by means of a SCCR. The solution is adopted in different degrees of diffusion in European cities such as Antwerp, Florence, Lonato, Pisa, Santiago de Compostela, etc. In this paper, the Florence SCCR has been analyzed as a major use case describing the data, the services and the workflow. For data aggregation, the Km4City multi-ontology and tools have been used to collect data from GIS, utilities, open data, IoT networks, external services, social media, etc. The first Florence SCCR dashboard appeared in 2017 as a prototype, and, since that time, a great evolution has been occurred and the amount and type of data have increased. Most of the data are public and accessible on dashboards from the Web site of Snap4City.org, while a number of dashboards are private to the municipality. The results presented have shown the data regarding the last year of high usage of the platform. During this period, a particular attention by the users, on general overview aspects, mobility and the environment have been registered. In summary, a total of more than 6000 accesses have been made and 340,000 min have been spent on the major dashboards for those major areas in the year 2019, which is an average of 57 min per access.
